# *Eruca sativa* seed napin structural insights and thorough functional characterization

**DOI:** 10.1038/s41598-021-02174-6

**Published:** 2021-12-15

**Authors:** Binish Khaliq, Sven Falke, Qamar Saeed, Muhammad Bilal, Aisha Munawar, Arslan Ali, Gunnar Baermann, Habib-ur-Rehman Athar, Seema Mahmood, Christian Betzel, Qurban Ali, Ahmed Akrem

**Affiliations:** 1 Botany Division, Institute of Pure and Applied Biology, Bahauddin Zakariya University, Multan, Pakistan; 2 Botany Division Institute of Molecular Biology and Biotechnology, The University of Lahore, Lahore, Pakistan; 3 Laboratory for Structural Biology of Infection and Inflammation, The Hamburg Centre for Ultrafast Imaging, University of Hamburg, c/o DESY. Build. 22a, Notkestrasse 85, 22607 Hamburg, Germany; 4 Department of Entomology, Bahauddin Zakariya University, Multan, Pakistan; 5 Centre for Applied Molecular Biology, University of Punjab, Lahore, Pakistan; 6 Department of Chemistry, University of Engineering and Technology, G.T. Road, Lahore, 54890 Pakistan; 7 Dr. Panjwani Center for Molecular Medicine and Drug Research, International Center for Chemical and Biological Sciences, University of Karachi, Karachi, Pakistan; 8 Molekulare Phytopathologie, Universität Hamburg, Biozentrum Klein Flottbek Ohnhorststr, 1822609 Hamburg, Germany

**Keywords:** Biochemistry, Proteins

## Abstract

A potent napin protein has been thoroughly characterized from seeds of rocket salad (*Eruca sativa*). *Eruca sativa* napin (*Es*Nap) was purified by ammonium sulfate precipitation (70%) and size-exclusion chromatography. Single intact 16 kDa *Es*Nap band was reduced to 11 and 5 kDa bands respectively on SDS-PAGE. Nano LC–MS/MS yielded two fragments comprising of 26 residues which showed 100% sequence identity with napin-3 of *Brassica napus*. CD spectroscopy indicated a dominant α-helical structure of *Es*Nap. Monodispersity of *Es*Nap was verified by dynamic light scattering, which also confirmed the monomeric status with a corresponding hydrodynamic radius of 2.4 ± 0.2 nm. An elongated ab initio shape of *Es*Nap was calculated based on SAXS data, with an R_g_ of 1.96 ± 0.1 nm. The ab initio model calculated by DAMMIF with P1 symmetry and a volume of approx. 31,100 nm^3^, which corresponded to a molecular weight of approximately 15.5 kDa. The comparison of the SAXS and *ab* initio modeling showed a minimized χ^2^-value of 1.87, confirming a similar molecular structure. A homology model was predicted using the coordinate information of *Brassica napus* rproBnIb (PDB ID: 1SM7). *Es*Nap exhibited strong antifungal activity by significantly inhibiting the growth of *Fusarium graminearum*. *Es*Nap also showed cytotoxicity against the hepatic cell line Huh7 and the obtained IC_50_ value was 20.49 µM. Further, strong entomotoxic activity was experienced against different life stages of stored grain insect pest *T. castaneum*. The result of this study shows insights that can be used in developing potential antifungal, anti-cancerous and insect resistance agents in the future using *Es*Nap from *E. sativa*.

## Introduction

Plants are facing various pathogenic organisms in their environment, i.e. bacteria, fungi and insects. As a result plants can produce a large variety of antimicrobial compounds such as phytoalexins and proteins^1,2^. In particular, plants protect themselves by secretion of small antimicrobial or antifungal proteins such as lipid transfer proteins^3^, snakins^4^, plant defensins^5^, hevein-like peptides^6^, glycine-rich peptides and napins^7^. Napin (2S albumin) is a low molecular mass protein present in *Brassicaceae* oilseed and belongs to the prolamin superfamily^8^. Among *Brassicaceae* seed storage proteins (SSPs), napin is the second most abundant protein after cruciferin and constitutes approx. 15–45% of all SSPs, depending on the specific species.

*Brassica napus* napins are heterodimers consisting of two polypeptides^9^ bridged through inter-chain covalent disulfide linkages^10^. Molecular structure determinations of napins revealed that disulfide bonds formed by cysteine residues play a crucial role. Cysteine residues are considered as conserved features of napins, due to their location and number in the polypeptide chain. There are eight cysteine amino acids, together called “eight Cys motif”, that have been pinpointed in different Napin isoforms; six in the long chain and two in the short chain^9^. Antimicrobial proteins of plants can be used to treat antibiotic-resistant microorganisms^11^. Further, plant-derived antimicrobial compounds are of high clinical value for the treatment of bacterial infections and infections caused by several fungi as well^12^. Specifically, some napins possess cytotoxic effects, whereby they can be applied in the development of new anti-cancer drugs^13^. Napin genes are being used in the development of transgenic plants expressing higher levels of napins, making them more pathogen resistant supporting a reduction of yield losses in the agriculture sector^14^. In short, the promising bioactivities of napins make them suitable candidates to act against a number of human pathogens^15,16^.

Rocket Salad (*Eruca sativa* Miller), locally known as Taramira; is an annual herb and belongs to the family *Brassicaceae* (*Cruciferae*). It is grown in different parts of the Indo-Pak subcontinent and in the Middle East. *Eruca sativa* is a minor oil crop; widely used as culinary and for medicines as remedies for different diseases. There is only sporadic information available about phytochemistry and bioactivity of this oily crop^17^. The regular consumption of *E. sativa* has been associated with the prevention of cardiovascular diseases and reduction in cancer risk^18,19^. It is known to have diuretic and anti-inflammatory activities^20^. *Eruca* seeds possess various proteins, glucosinolates, vitamins A and C, flavonoids, erucic acid and a relatively high oil content^17,21^. It is commonly used as animal feed in Asia, particularly in India and Pakistan. In view of its potential medicinal uses, it is hypothesized that Rocket Salad might have antimicrobial proteins/peptides in their seeds/leaves which can be exploited for the development of anti-cancerous drug after detailed understanding of its structure with subsequent characterization. Present study describes the structural insights and thorough functional characterization of a napin, which was identified and purified from seeds of *E. sativa*.

## Results

### Napin purification

**Figure 1:**
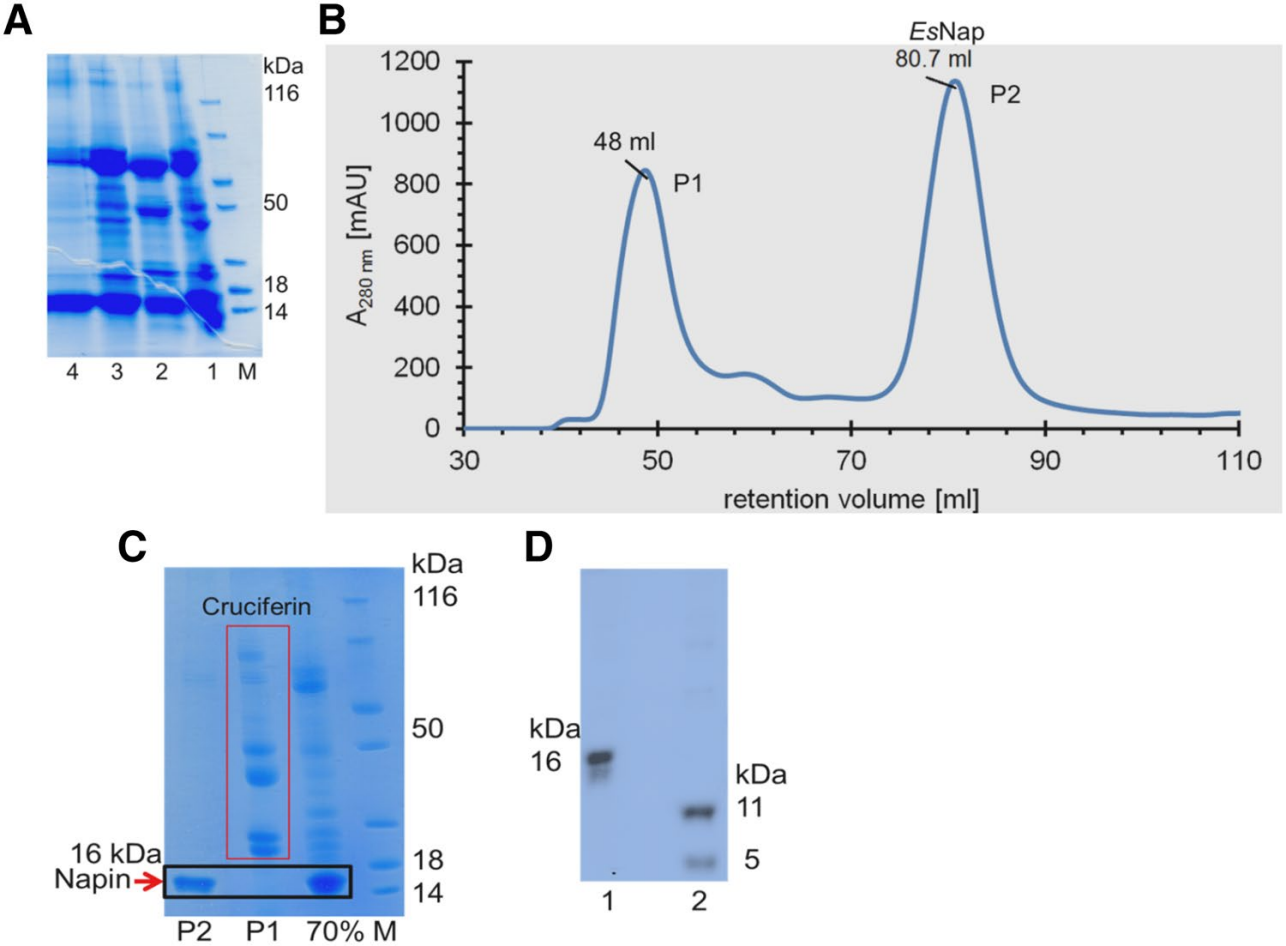
Purification and molecular weight determination of *E. sativa* seed napin (*Es*Nap). (**A**) Partial purification of *Es*Nap from crude extract, lane 1; crude extract, lane 2 and 3; 50% ammonium sulfate saturation of crude extract supernatant, lane 4; re-dissolved and dialyzed 70% ammonium sulfate saturation pellet, M; Protein Ladder (Catalog, 22,610). (**B**) Purification of *Es*Nap by calibrated size exclusion chromatography (SEC, HiLoad 16/60 Superdex 75) subsequent to ammonium sulfate precipitation (70% saturated solution). Chromatogram showed the P1 and P2 with retention volume of 48 and 80.7 ml respectively. (**C**) SDS-PAGE showed that P1 has cruciferin and *Es*Nap was found in P2 of the chromatogram while lane 1 is the 70% solubilized pellet before SEC. (**D**) SDS-PAGE analysis of napin under non-reducing condition (lane 1) and in the presence of 20 mM DTT (lane 2) which splitted the intact band into approximately 11 and 5 kDa respectively.

Napin was precipitated by ammonium sulfate from the crude extract of *Eruca sativa* (Fig. 1A). Napin protein remained in supernatant after 50% (w/v) (NH_4_)_2_SO_4_ saturation constant while subsequent 70% precipitated the protein in pellet. The dissolved pellet was extensively dialyzed to remove any further salt traces and subjected to size-exclusion chromatography to obtain the highly purified protein fractions. Ultimately, an optimized combination of ammonium sulfate precipitation along with chromatographic steps provided a > 95% pure napin solution from seeds of *Eruca sativa*, as judged by SDS-PAGE analysis. The gel filtration chromatogram showed two absorbance peaks and the corresponding SDS-PAGE showed that first peak contained high molecular weight cruciferins while *Es*Nap was found in the second peak (Fig. 1B,C). *Es*Nap fractions with maximum purity were stored at 4 °C. Further SDS-PAGE analysis showed the splitting of napin (16 kDa) into two daughter fragments of 11 and 5 kDa upon addition of DTT confirming the quaternary structure as well as the presence of inter-chain disulfide bonds within the structure (Fig. 1D). The purification strategy resulted in purification of 13-fold with a 7.5% yield (Table 1) from one gram of *E. sativa* seed powder.

**Table 1:** Purification steps of *Eruca sativa* Napin (*Es*Nap) from one gram of seed powder.

Purification steps	Total protein (mg)	Purification (times)	Recovery (%)
Crude extract	80	1	100
Ammonium sulfate fractionation (50% supernatant)	54	1.48	67
Ammonium sulfate fractionation (70% pellet)	20	4	25
Hi Load 16/60 Superdex 200 column	6	13	7.5

### Protein identification

**Figure 2:**
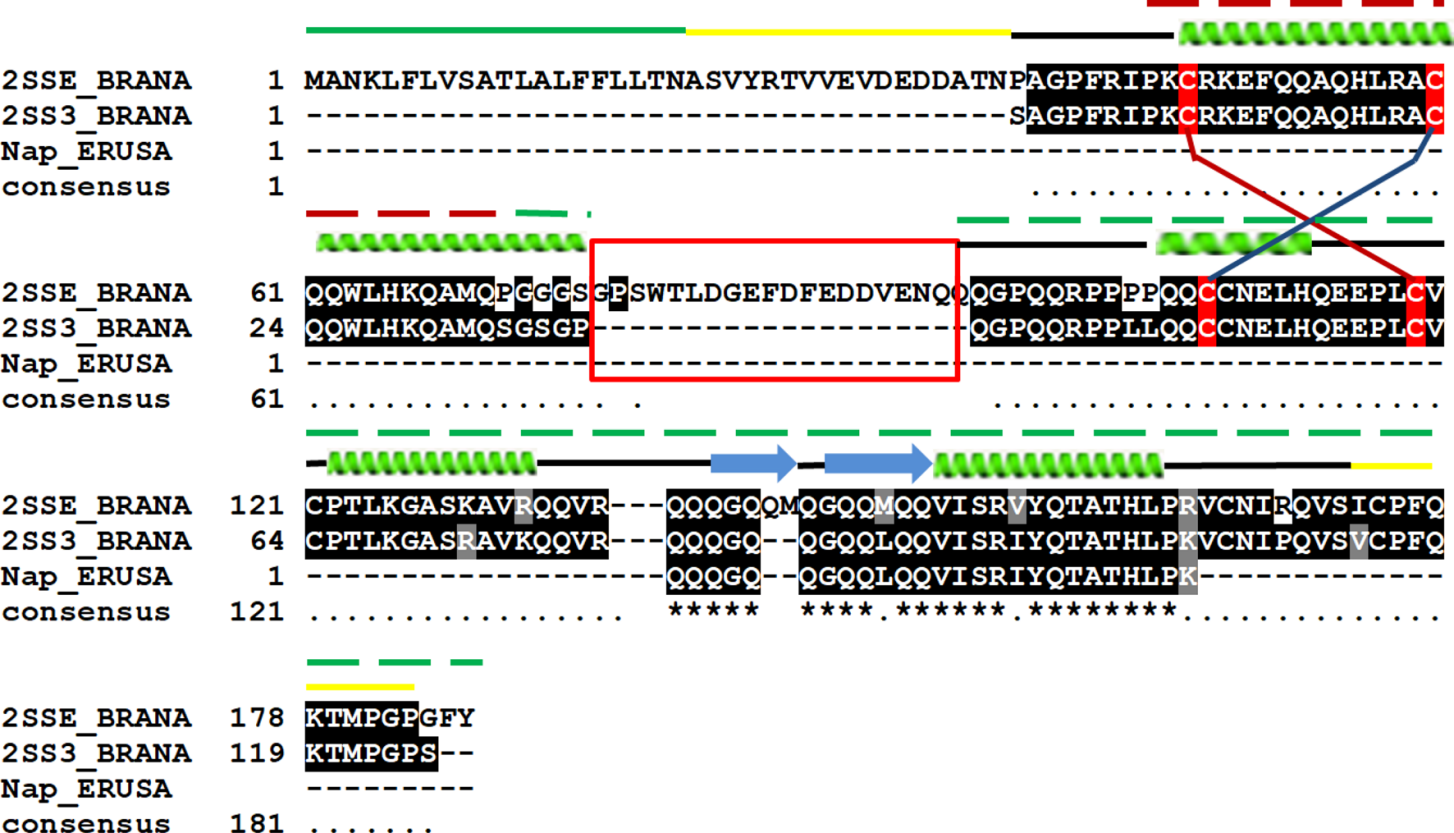
Multiple sequence alignment of *EsNap* with other closely related plant napins 2SS3_BRANA (*Napin-3: Brassica napus*)*,* 2SSE_BRANA (*Napin embryo-specific: Brassica napus*) and Nap_ ERUSA (Napin: Eruca sativa). Secondary structure elements (α-helices and β-sheets) of EsNap are indicated at the top. Signal peptide, N/C terminus and turns are indicated with green, yellow and black bars respectively. Identically conserved residues are labeled by asterisks (*), while semi-conserved substitutions are labeled by single dots (.) and cysteine involved in disulfide bonds between two chains are shown in red color. The highly flexible region is highlighted with a red box. Smaller and larger chains are shown in dot line (–) upper on the secondary structure elements in green and red color respectively Multiple sequences alignment was performed by using ClustalW in the default set up and BoxShade server.

LC–MS/MS identified two peptides (IYQTATHLPK^10^, QQQGQQGQQLQQVISR^16^) (peak raw data is shown in Fig. S1: Supplementary material) which showed 100 and 85% sequence identity with napin-3 (UniProtKB ID: P80208) and embryo-specific napin (UniProtKB ID: P09893) from *Brassica napus*, respectively. The fragmented sequence of *Es*Nap was used for multiple sequence alignment with napin-3 and embryo-specific napin of *Brassica napus* (Fig. 2)*.* The alignment analysis showed that *Es*Nap is more identical to *B. napus* napin.

### Isoelectric focusing (IEF) of EsNap

The result of isoelectric focusing showed that *Es*Nap is basic protein and have the basic pI. A single band observed after isoelectric focusing (IEF) reveals the basic pI of 8.0, like other already reported napins from *Brassicaceae* species (Fig. S2: Supplementary material).

### Secondary structure determination of EsNap

**Figure 3:**
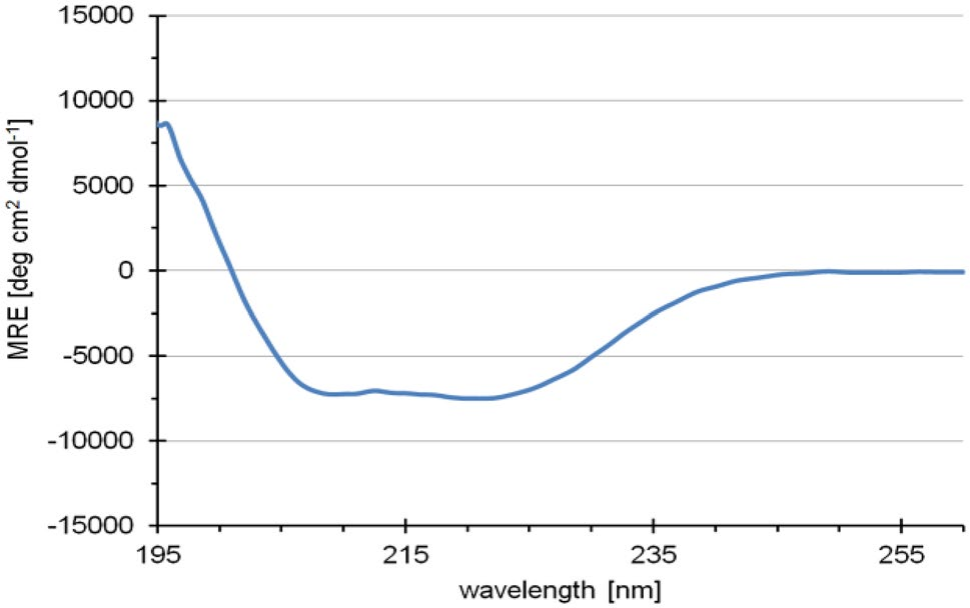
CD spectroscopy of *Es*Nap. Far-UV CD spectrum of *Es*Nap is indicating predominantly α-helical secondary structure (38%).

The circular dichroism (CD) spectrum (Fig. 3) showed predominantly α-helical structure, as indicated by two distinct ellipticity minima^22^, as well as some flexible loops. The CD spectrum corresponds to approx. 38% α-helix, 9% β-sheet, 19% turn and 34% random coil structure applying the software Secondary Structure Estimation (Jasco) and the algorithm according to Yang et al. for data interpretation^23^ and fit curve calculation.

### DLS analysis and assessment of tertiary and quaternary structure by small-angle X-ray scattering

**Figure 4:**
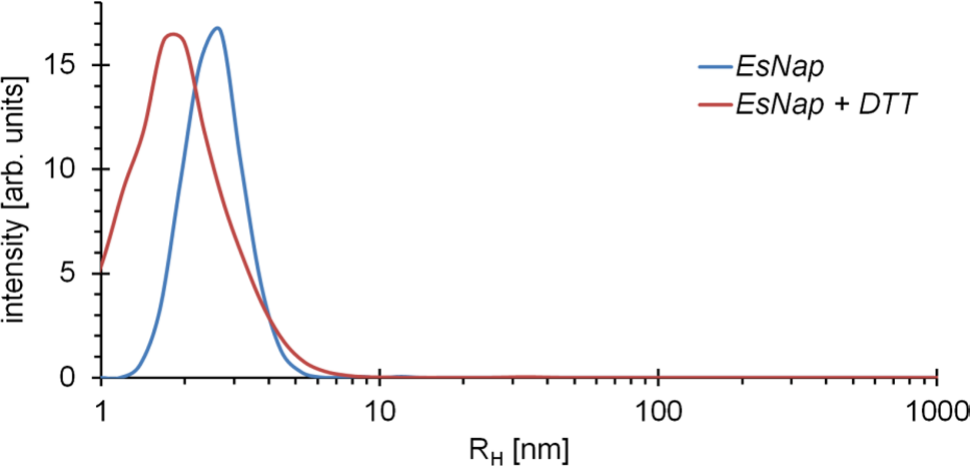
The particle size distribution obtained by dynamic light scattering reveals the monodispersity of the *Es*Nap solution (blue line) with a hydrodynamic radius of 2.4 ± 0.2 nm. A broadening and shift in hydrodynamic radius (1.8 ± 0.2 nm; red line) was observed after the addition of 20 mM DTT to *Es*Nap solution which confirmed the splitted banding pattern as already observed on SDS-PAGE under reduced conditions. Obtained results indicated that *Es*Nap is a monomeric globular protein.

**Figure 5:**
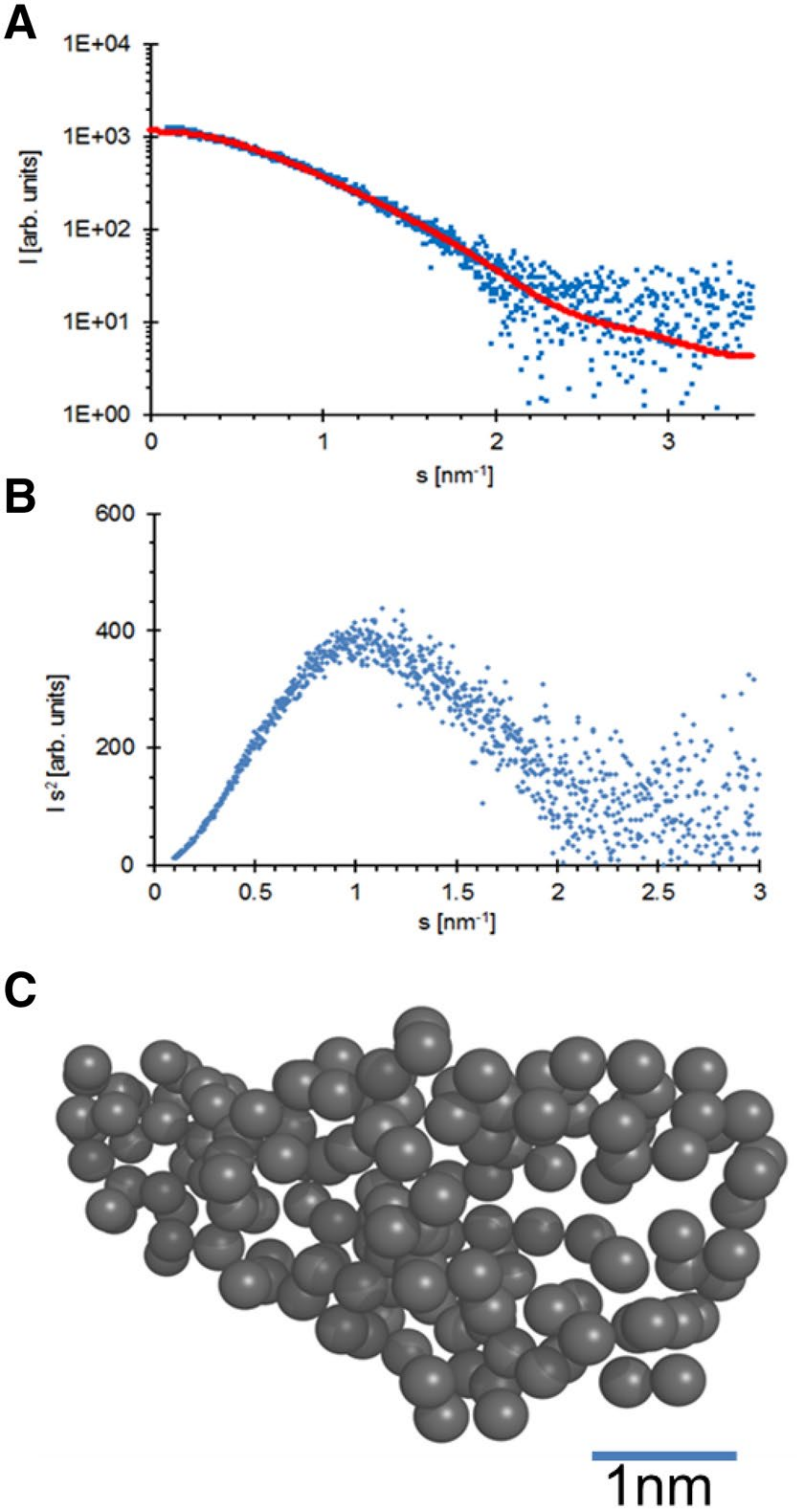
(**A**) Small-angle X-ray scattering intensity plot of pure *Es*Nap and the corresponding calculated fit curve (red) resembling a single ab initio model as displayed in panel C and calculated by GASBOR. The plot shows the dependency of the scattering intensity I on the momentum transfers. (**B**) Kratky plot of the scattering intensity distribution indicating a compact and relatively rigid protein structure (**C**) Ab initio model possessing P1 symmetry; the scale bar is 1 nm in length.

Monodispersity and homogeneity of *Es*Nap solution was verified by applying DLS calculation. A hydrodynamic radius of 2.4 ± 0.2 nm is (Fig. 4) indicating that the protein is monomeric in solution. The averaged scattering amplitudes of *Es*Nap are indicating an R_g_ of 1.96 ± 0.01 nm according to the Guinier approximation determined by AUTORG, which is implemented in PRIMUS^24^. The P(R) function is indicative for an oblate particle with a maximum diameter of 5.6 nm. According to the volume of correlation, the molecular weight of napin is approximately 16 kDa. Considering P1 symmetry of a monomer, an ab initio model was calculated using GASBOR, with a corresponding molecular weight of 15 kDa. The particle shape is rather oblate with extended C- and N-terminus that may harbor a certain degree of flexibility, as indicated by superimposition (Fig. 5). The molecular weight estimation based on SAXS data well supports complementary results of DLS and electrophoresis experiments.

### EsNap homology modeling

**Figure 6:**
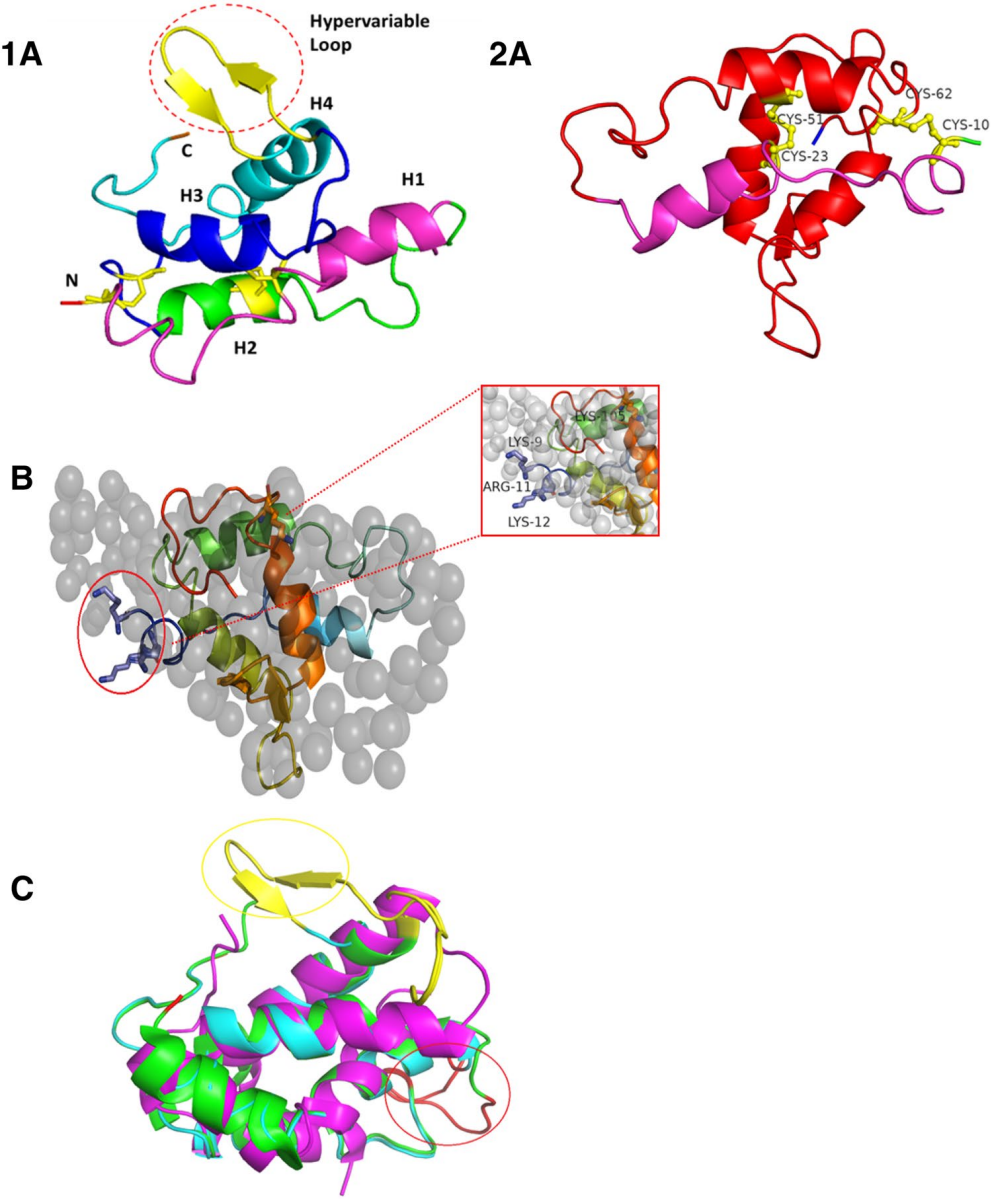
Overall structure of *Es*Nap *in-silico* model and structural alignments. (**1A**) The 3D structure was predicted by homology modelling and is shown as ribbon diagram. The 3D model of *Es*Nap consisted of four helices and a hypervariable loop comprising of two antiparallel β-sheets (yellow color) marked by red circle. (**2A**)The smaller chain composed of N-terminal (green) to H1 helix (purple) and longer composed of H2, H3, H4 and two shortβ-sheets (red). The disulfide bridges are represented by yellow spheres (**B**) Ab initio model of *Es*Nap with P1 symmetry (grey spheres), Lys 9, Arg 11, Lys 12 (blue) and Lys 105 (red) form flexible N and C-terminal of ab initio model and 3D model of *Es*Nap have been superimposed; involved in antifungal and anticancer activity. (**C**) Structural alignment of *E. sativa* napin (Cyan, *Es*Nap) with BnIb from *Brassica napus* (Green, pdb code: 1SM7) and 2S albumin from *Moringa oleifera* (Purple, pdb code: 5DOM). Hypervariable loops and connecting loops between two polypeptide domains are colored in yellow and red, respectively.

The *Es*Nap 3D model consists of a globular four-helix motif with up and down topology for the predicted structure where the first helix (H1) is not splitted into two helices like in rproBnIb and in rRicC3. Structures rproBnIb and rRicC3 have the five –helix from residues 3–11 (helix Ia), 16–25 (helix Ib), 44–54 (helix II), 57–71 (helix III), and 81–95 (helix IV)^25^. Two disulfide bridges (CYs10-Cys62 and Cys23-Cys51) are formed between the smaller and longer chains in Fig. 6(2A). H3 and H4 are almost antiparallel to each other and are connected by two short β-sheets, which are constituted by residues Gln76-Gln91, known as the hypervariable region in 2S albumins^26–28^, because of the high variability in length and sequence composition as shown in Fig. 6(1A). The scattering amplitudes of *Es*Nap processed by PRIMUSQT were compared to the predicted 3D model of *Es*Nap using the program CRYSOL, as shown in Fig. 6B with a calculated minimized χ^2^-value of 1.87. The manual superimposition well confirms the conclusion that the structures are widely similar, including the molecular weight comparison with the ab initio model. Lys 9, Arg 11, Lys 12 (blue) and Lys 105 (red) residues form flexible N and C-terminal of *Es*Nap ab initio model are as shown in Fig. 6B. These residues are involved in the antifungal and anticancer activity of *Es*Nap. The in silico model of *Es*Nap was aligned with the NMR structure of the pronapin precursor, BnIb from *Brassica napus* (PDB-ID: 1SM7) and crystal structure of 2S albumin from *Moringa oleifera* (PDB ID: 5DOM) and the respective RMSD values only for the carbon alpha (109) superposition are 0.89 Å and 3.37 Å respectively (Fig. 6C). The lower RMSD value was observed with the recombinant pronapin structure, which showed that the *Es*Nap structure is structurally highly similar to the pronapin precursor from *Brassica napus*. The high RMSD value was obtained for 2S albumin from *M. oleifera* due to presence of a significant variable loop in the form of antiparallel small β sheet in *Es*Nap 3D structure marked by red circle and other loop areas (Fig. 6A).

### Antifungal activity

**Figure 7:**
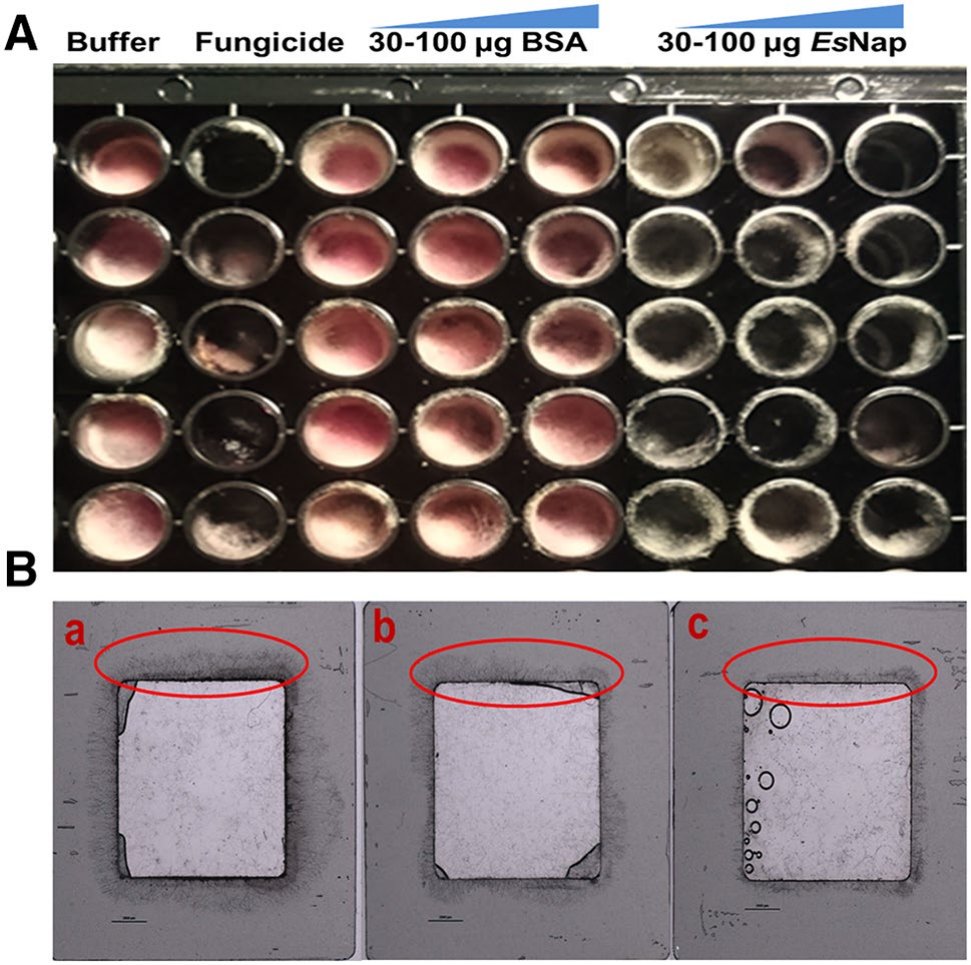
Inhibitory activities of *Es*Nap towards growth of *Fusarium graminearum* mycelia. (**A**) All the three *Es*Nap concentrations (30, 50 and 100 µg) in medium inhibited the fungal growth, while regular and optimal fungal growth was observed in medium mixed with buffer and 30–100 µg BSA as well as only in buffer as negative control. No fungal growth was observed in the medium with fungicide TOPSIN as positive control. (**B**) Inhibition of mycelia growth of *Fusarium graminearum* in gene frame chamber. [**a**: BSA (100 µg); **b**: phosphate buffer; **c**: Concentrated *Es*Nap protein (100 µg)].

An antifungal activity of *Es*Nap was observed against *Fusarium graminearum* as shown in Fig. 7A. All *Es*Nap concentrations of 30, 50, and 100 μg inhibited the fungal mycelia; growth after 96 h of incubation however, 50 and 100 µg *Es*Nap exhibited 50% inhibition of the mycelia growth after 96 h (Fig. S3: Supplementary material). BSA was provided as an alternative protein nutrition source for comparison. There was significant inhibition of conidia germination with the 100 µg *Es*Nap in gene frame experiment as shown in Fig. 7B. The conidia germinate over night at 26 °C and were imaged the next morning.

### Cell cytotoxicity assessment

**Figure 8:**
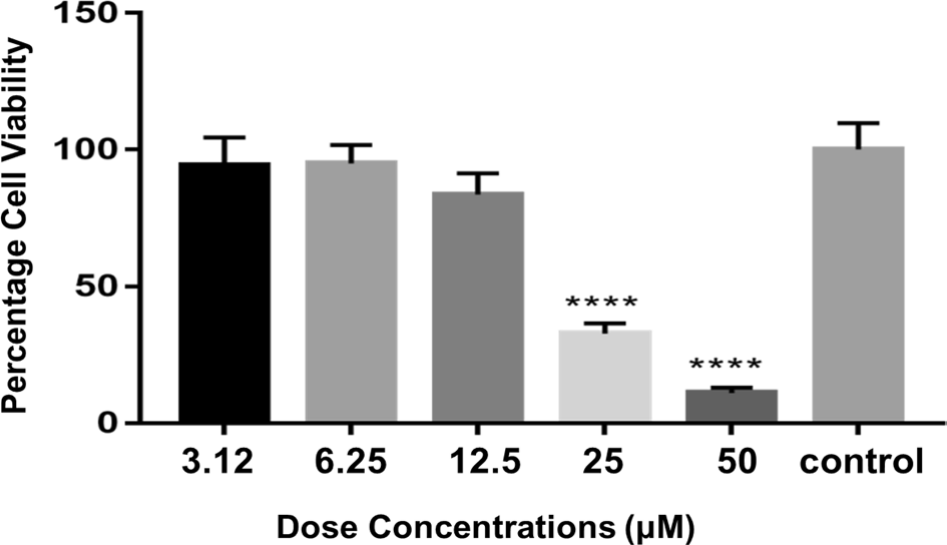
Effect of different doses of *Es*Nap on Huh-7 cells visualized by plotting the protein concentration (µM) against the cell viability (%). Asterisks are indicating significant mycelia inhibition at concentrations of 25 and 50 µM; however, calculated IC_50_ value of *Es*Nap was 20.49 µM.

The number of living cells is represented by the absorbance of soluble formazan in the visible light spectrum^29^. The cytotoxic effect is shown in Fig. 8. Assessment of ell viability of Huh-7 cells against different dose concentrations by MTT assay. The results are expressed as mean ± SD (n = 3). ****P < 0.0001 is observed at doses of 25 and 50 µM. The IC_50_ value of *Es*Nap was 20.49 µM calculated by non-linear regression analysis (Fig. S4: Supplementary material).

### Entomotoxicity assessment

Purified *Es*Nap significantly inhibited the development of *T. castaneum* populations at all stages. Different biological traits i.e. number of larvae, male and female pupae including adults and total population were monitored. Results clearly revealed the effectiveness of the napin flour mixture when fed to *T. castaneum.* The results obtained at all protein concentrations are highly significant when compared to the control. The highest larval population was observed in the control group, i.e. 130.8 ± 10.0 with no napin provided, while it is obvious that all protein concentrations reduced the number of larvae significantly (*P* < 0.0006, F = 9.96) (Table 1). Similarly, the total numbers of counted pupae and adults were strongly reduced at all concentrations of *Es*Nap and a maximum was recorded in the control with no *Es*Nap treatment (86.6 ± 10.5 and 41.0 ± 8.0) (*P* < 0.0, F = 22.5). In parallel, average larval, pupal and adults populations were decreased with increasing concentration, which means the smallest populations were observed at 3 mg/ml concentration followed by 2 mg/ml and 1 mg/ml of *Es*Nap. The ratio of male and female pupae as well as adults was also recorded; the male population was significantly larger in number as compared to the female population for all *Es*Nap concentrations (Table 2).

**Table 2:** Survival of *T. castaneum* in the presence of different *Es*Nap concentrations.

Concentration	Total larvae	Male pupae	Female pupae	Total pupae	Male adults	Female adults	Total adults
3 mg/ml	39.4 ± 2.1c	10.8 ± 1.5c	9.4 ± 1.8b	22.2 ± 1.2c	4.8 ± 1.2c	4.6 ± 0.7b	9.4 ± 1.9b
2 mg/ml	47.0 ± 1.6b	13.0 ± 4.0b	9.4 ± 3.0b	22.0 ± 3.2c	6.4 ± 0.7b	5.0 ± 0.4b	11.4 ± 0.9b
1 mg/ml	51.8 ± 2.5b	14.4 ± 2.2b	10.4 ± 2.0b	28.8 ± 2.8b	7.4 ± 0.4b	6.2 ± 0.6b	13.6 ± 1.4b
Negative control	130.8 ± 10.0a	45.6 ± 4.2a	41.0 ± 7.0a	86.6 ± 10.5a	23.4 ± 3.8a	17.6 ± 4.7a	41.0 ± 8.0a

Least significant differences at the 1% probability level (*P* < 0.01).

## Discussion

Napins are present in leaves, seeds, roots and stems of a number of plant species belonging to cereals and crucifers. Napins are synthesized as larger precursors, which have a post-translational N-terminal signal peptide and a C-terminal precursor peptide. Napin from the seeds of Taramira (*Eruca sativa*), a member of the family *Brassicaceae*, has been isolated and characterized. *Eruca sativa* napin (*Es*Nap) has a molecular mass of around 16 kDa, as determined by SDS-PAGE. Most of the napins known today have molecular masses in the range of 16 kDa and showed relatively high levels of sequence similarity. Napins are polypeptides containing S–S bonds that are formed under reducing conditions. Four S–S bonds existed in native napin structures, two between the chains and two within the larger chain^30–32^. Napin family is typically rich in arginine, lysine, and cysteine residues and have a strong antimicrobial activity^33^. *Es*Nap protein has basic properties due to high level of arginine and lysine compared to other amino acids. The IEF of *Es*Nap showed the pI value of approximately 8.00. In 1981, Crouch and Sussex indicated that napins have basic pI of 11 which is verified due to high number of arginine, lysine and histidine residues^34^.

CD showed that *Es*Nap has highly helical secondary structure content. In this context, Sharma et al.^35^ reported that 2S Albumin (napin) from seeds of *Wrightia tinctoria* has a high content of α-helices. Previous studies have reported 40–45% helices and 16–20% β-sheets, 25% α-helix and 38% β-sheets for napin. The high content of α-helix in napins may promote some toxic biological activities against pathogens and facilitate the dynamic association of the protein with membranes, as summarized for some peptides by Bechinger^36^. Additional structural information about *Es*Nap was obtained by SAXS analysis which strongly indicated that *Es*Nap is globular with slightly elongated native form in solution and exists in a monomeric state. The shape factor, *R*_g_ divided by *R*_H_ as determined by DLS, has a value of 0.8, which is indicative for a globular and slightly elongated shape of *Es*Nap particles in agreement with the displayed ab initio model shown in Fig. 5C. N and C-terminal basic residues (Lysine and Arginine) of *Es*Nap protein formed the flexible part of ab initio model in Fig. 6B and responsible for antifungal and anticancer activity in *Es*Nap due to the distribution of cationic charge.

*Es*Nap 3D structure has α-helix dominant secondary element as well as possesses high lysine and arginine contents and these properties together are responsible for antifungal and anti-cancerous activities. It has already been reported that an amphipathic conformation and high cationic charge distribution are responsible for antifungal activity of napins^37^. *Es*Nap 3D structure showed strong amphipathic behavior parallel to their high lysine and arginine content, and thus fully comply with these two requirements. Amphipathic α-helix structure of *Es*Nap may be involved in the CaM (Calmodulin) antagonist and formation of pores in membranes. This is probably because CaM and two subunits of *Es*Nap contain similar α-helical conformations. Neumann and his colleagues reported that amphipathic α-helix structure of napins showed the CaM (Calmodulin) antagonist activity and may be involved in development of membranous pores^38^.

*Es*Nap exhibited antifungal activity against *Fusarium graminearum* at 30–100 µg quantity. Initially, (48–72 h) as shown in figure S4, the fungal growth was promoted by the napin protein itself which is presumably due to the fact that the fungus partly metabolized the protein and used it as a carbon or nitrogen nutrition source. It is well known that nitrogen is an essential requirement for growth, and the ability to metabolize a wide variety of nitrogen sources enables fungi to colonize different environmental niches and survive nutrient limitations^39^. However, later on 96–120 h, napin-treated samples showed a significant reduction in growth compared to BSA-treated-samples (Fig. S4). Tomar and his colleagues reported that pumpkin 2S albumin inhibited the growth of *Fusarium oxysporum*, *Phanerochaete chrysosporium* and *Aspergillus flavus* grown in PDA medium at 50 and 100 µg protein dissolved in a similar culture volume^40^. Napin (PR protein-13) from *Pennisetum glaucum* (pearl millet) inhibited the growth of *Sclerospora graminicola* spores at a quantity of 100 µg^41^. Wheat puronapins showed antifungal activity against *Rhizoctonia solani* by membrane permeabilization*,* responsible for significant crop losses and rice sheath blight^42^.

Plant napins are cytotoxic and have shown anti-cancerous activities. *Es*Nap showed the cytotoxic activity against the Huh7 cells. *Es*Nap concentrations of 25 and 50 µM showed the significantly killing of cancerous cells and IC50 was obtained at 20.49 µM by non-linear regression as shown in the Fig. 8. *Es*Nap could be potential candidate for the development and formation of anticancer drug. Structural information of these proteins can be utilized for the design of new anticancer drugs. The 2S albumin from seeds of pumkin showed the cytotoxicity against the breast cancer cell line MCF-7, ovarian teratocarcinoma cell line PA-1, prostate cancer cell line PC-3 and DU-145 and liver hepatocellular carcinoma (Hep G2) (Tomar et al., 2014). Different concentrations of this protein (1, 5, 10, 20, 30 and 40 µM), were used against cell lines. In response to 20 µM of protein, the cell viabilities of breast cancer (MCF-7), ovarian teratocarcinoma (PA-1), prostate cancer (PC-3 and DU-145) and hepatocellular carcinoma (Hep G2) by treatment were found to be 43.40%, 54.81%, 49.12%, 43.3% and 45.69% respectively^40^.

Due to the alarming situation regarding the impact of conventional insecticides on human health and the surroundings, the search for novel molecules with insecticidal activity with minimal adverse effects has become paramount^43^. Purified *Es*Nap produced strong negative effects on all life stages of stored grain insect pest *T. castaneum*. The plant extracted protein PA1b from *Pisum sativum* was first reported to act against stored grain insect pests especially on cereal weevils and was found to be a valuable naturally occurring biopesticide^44,45^. Many toxic metabolites with antimicrobial activity released by plants are currently commercially available as they also show an individual level of toxicity towards insect pests. Consequently, entomo-toxic plant compounds are an appreciated starting point to further develop bio-insecticides against stored product insect pests^46^ on a long-term scale after carefully verifying their respective persistency and toxicity spectrum. Muench et al.^47^, characterized pea albumin PA1b and mapped its binding site on insect vacuolar ATPase. Its interaction is influencing the toxicity and a similar mechanism is conceivable in case of napins resulting in reduced *T. castaneum* populations, as also observed by Da Silva and his group in 2012. The saponin 3-GlcA-28-AraRhaxyl-medicagenate from *Medicago truncatula* seeds due to its high toxicity against *Sitophilus oryzae* (Da Silva., 2012). This 3-GlcA-28-AraRhaxyl-medicagenate exhibits repellent properties and has a CMC of about 0.6 mM. The exposed protein treatment to the insect is actually 0.4 mg/g of the flour which is not that high and review of literature supports our treatment values. However, there could be many reasons for toxicity of napins against *T. castaneum* as described previously^47–50^. Experiments applying napin of *Pyrularia*, which is hemolytic, cytotoxic and neurotoxic, suggest that negatively charged membrane lipids are targeted directly by conserved basic amino acids^51^. Consequently, it is concluded that napins commonly do not possess receptor specificity but induce the formation of oligomeric protein-lipid complexes and ion permeable membrane pores^52,53^. This mechanism would target a broad spectrum of species and is in agreement with the observed toxicity of *Es*Nap towards the species that were selected for this study. Nonetheless, an improved understanding of the structure–function relationship and mode of action is essential for understanding the ecological mechanisms promoted by plant napins as well as utilizing napins for more biotechnological and medical applications.

## Experimental

### Plant material

Fresh seeds of *Eruca sativa* were obtained from Botanical garden; Bahauddin Zakariya University, Multan. *Eruca sativa* seeds were identified morphologically (color and shape) by the Department of Plant Protection, Government of Pakistan, Multan. It have been confirmed that the experimental samples of plants, including the collection of plant material, complied with relevant institutional, national, and international guidelines and legislation with appropriate permissions from the Department of Plant Protection, Government of Pakistan, Multan for collection of plant specimens.

### Purification of napin

Seeds (5 g) of *E. sativa* were ground by mortar and pestle to a powder form. The powder was homogenized in 100 ml of 100 mM phosphate buffer (pH 7.0) containing 1 mM phenylmethylsulfonyl fluoride (PMSF) as protease inhibitor. The mixture was stirred continuously for three hours at 25 °C. After stirring, the sample was centrifuged (Ogawa 6470) at 10,000×g for 30 min and the pellet was discarded. The supernatant was filtered through Whatman filter paper (pore size 8 µm; EW-06648-46) to remove any particulate matter. The clear crude extract (90 ml) was subjected to 50 (supernatant) and 70% ammonium sulfate precipitation by stepwise slow addition of salt with constant stirring using a magnetic stirrer at 4 °C. The precipitated proteins were separated at low centrifugation speed of 3000 rpm for 4 min. The supernatant was removed and the resulting pellet was re-dissolved in 10 ml of the same phosphate buffer and dialyzed thoroughly in 100 mM phosphate buffer (pH 7.0) at 4 °C with gentle stirring. The desalted napin was further purified by injecting the solution onto a pre-equilibrated Hi Load 16/60 Superdex 200 column (GE Healthcare, ÄKTA prime plus). The protein was eluted with the same phosphate buffer at a flow rate of 1.0 ml/min. Absorbance of the eluents was recorded at a wavelength of 280 nm. The fractions with maximum protein content were analyzed and combined after running of 15% SDS-PAGE (E-VS10-SYS, omniPAGE mini-System, Germany)^54^. The concentration of purified protein was quantified by Nanodrop 2000c (Thermo Scientific, peqLab, Germany).

### Mass spectrometric analysis

Gel bands stained with colloidal Coomassie were cut out and reduced and alkylated with DTT (10 mM, 56 °C, 30 min.) and Iodoacetamide (IAA, 55 Mm, room temperature in dark), respectively. The protein in the gel was digested with trypsin (conditions: 5 ng trypsin/µl (sequencing grade modified trypsin, Promega, Madison, USA) in 50 mM NH_4_HCO_3_, 37 °C, 16 h). After digestion, the gel pieces were repeatedly extracted (65% acetonitrile/5% formic acid) the combined extracts were dried in a vacuum concentrator and redissolved in 20 µl 0.1% formic acid. LC–MS/MS measurements were performed by injecting the samples into a nano liquid chromatography system (Dionex UltiMate 3000) coupled via electrospray-ionization (ESI) to an orbitrap mass spectrometer (Orbitrap Fusion, Thermo Scientific, Bremen, Germany). The samples were loaded (3 μl/min) onto a trapping column (Acclaim PepMap μ-precolumn, C18; buffer A: 0.1% formic acid in H2O; buffer B: 0.1% formic acid in acetonitrile) with 2% buffer B, washed for 5 min with 2% buffer B (3 μl/min) and the peptides were eluted (300 nl/min) onto the separation column (Acclaim PepMap 100, C18, 75 μm × 250 mm, gradient: 2–30% B in 35 min). Mass spectrometric analysis was performed in positive ion mode. LC–MS/MS analysis was carried out in data dependent acquisition mode (DDA). MS ions were detected in orbitrap at 120 k resolution while MS/MS spectra were recorded in the ion trap as detector.

LC–MS raw data were processed with Proteome Discoverer 2.0 (Thermo Scientific, Bremen, Germany). For identification, MS/MS spectra were searched with Sequest HT against the *Arabidopsis* and the plant Uniprot database (https://www.uniprot.org, downloaded November 10, 2019). The searches were performed using the following parameters: precursor mass tolerance 10 ppm, fragment mass tolerance 0.5 Da, two missed cleavages allowed, carbamidomethylation of cysteine residues as fixed modification, oxidation of methionine residues as a variable modification. Identifications were validated manually.

### Protein identification

For protein identification, a search for sequence similarities was performed applying a BLAST tool through feeding of residual sequences https://www.uniprot.org/blast. Homologous sequences were subsequently aligned using ClustalW https://www.genome.jp/tools-bin/clustalw in the default set up and BoxShade server https://embnet.vital-it.ch/software/BOX_form.html.

### Isoelectric focusing (IEF)

IEF was performed using 17 cm long and 0.5 mm thick gel strips (pH 3–10, Sigma). Purified protein (405 µg) was loaded onto the horizontal gel maintained at 28 °C in dehydration buffer containing 8 M urea, 2% CHAPS, 50 mM DTT, 0.2% Bio-Lyte ampholytes and 0.001% bromophenol blue overnight. The pI markers proteins (Sigma) ranging from 3 to 10 were co-electrophoresed to estimate the pI of the proteins under investigation. Isoelectric focusing was performed in an IEF focusing cell (Bio-Rad). The voltage was increased stepwise starting from 250 V for 20 min, 10,000 V for 2.5 h and 10,000 V for 12 h. The gels were maintained at 28 °C during the run. After IEF, the proteins were stained by Coomassie blue.

### Circular dichroism (CD) spectroscopy

CD spectroscopy experiments were performed to determine the secondary structure composition of napin applying a CD6 dichrograph instrument (Jobin Yvon, Longjumeau, France). Purified napin (0.2 mg/ml) was prepared in 25 mM phosphate buffer, pH 7.0 and the CD spectra of napin were recorded in the far-UV-range between 190 and 260 nm at 25 °C in a 1 mm path length quartz cell. A total of fifteen spectra were averaged after measuring the buffer separately. The percentage of secondary structure of napin was calculated by using Spectra manager™ software (Jasco).

### Dynamic light scattering (DLS)

Purified protein was analyzed by DLS using the SpectroLight 300 instrument (Xtal Concept, Germany) for confirming the monodispersity of the protein solution as well as the size distribution calculation of molecules.

### Small-angle X-ray scattering (SAXS)

Small-angle X-ray scattering data of napin at two different solution concentrations (3.2 and 6.5 mg/ml) were collected at EMBL beamline P12^55^ at the storage ring PETRA III (DESY, Hamburg, Germany). At a sample-detector distance of 3.0 m and a wavelength of λ = 0.124 nm, scattering data were collected applying a 2D photon-counting Pilatus 2 M pixel detector (Dectris) with the momentum transfer ranging from 0.03 nm^−1^ < s < 4.80 nm^−1^ (s = 4π sinθ/λ, where 2θ is the scattering angle). To exclude significant radiation damage, 20 successive X-ray exposures of napin of 45 ms each were compared and no significant changes in the intensity pattern were observed over time. Data were normalized to the intensity of the transmitted beam and radially averaged. The scattering pattern of the buffer was subtracted, and the difference curves were scaled for protein concentration. The radius of gyration Rg along with the particle pair-distance distribution function p(r), which further provides the maximum dimension Dmax, were computed by the automated SAXS data analysis pipeline SASFLOW and verified via PRIMUS^56^. Low resolution *ab* initio shapes of napin were generated based on the composite scattering curves applying the program GASBOR^57^. It uses an assembly of interconnected dummy residue spheres to generate a chain-like *ab* initio protein model that fits the experimental scattering data. The molecular weight of *Es*Nap was estimated by its excluded particle volume and further verified based on the forward scattering intensity of BSA (66 kDa; 5 mg/ml in 50 mM HEPES pH 7.5), which was measured in addition to verify beamline operation.

### Homology modeling and structure prediction

For the calculation of an *Es*Nap homology model, fasta sequence of napin-3 from *Brassica napus* (UniProtKB ID: P80208) was consequently used for the 3D modeling of *Es*Nap. Therefore, the primary sequence of napin-3 was subjected to model building via the Swiss-Model server^58–60^. The coordinate information of recombinant pronapin precursor, BnIb from *B. napus* (PDB-ID: 1SM7) was used as the most suitable template. The model was built based on the target-template alignment using ProMod3^61^. Coordinates of fragments with a conserved sequence comparing the target and the template were copied from the template to the model. Insertions and deletions were remodeled using a fragment library and side chains were then rebuilt. Finally, the geometry of the resulting model is regularized by using a force field. The images of the predicted model were prepared applying PyMOL^62^.

### Fusarium multi-well plate growth assay

The 96-well microtiter plate assay is a sensitive and fast method for large scale measurement of the inhibitory effect of antifungal substances in vitro. To determine the inhibitory impact of *Es*Nap on the germination and growth of the fungus *Fusarium graminearum*, the hypha growth over a time period of 120 h was obtained at an incubation temperature of 26 °C. Each well of a Greiner 96-well flat bottom plate contained 100 µl minimal medium^63^, 4 µl conidia suspension (125 conidia µl^−1^) of *F. graminearum* strain 8/1^64^ constitutively expressing GFP as well as *Es*Nap or BSA solution (30, 50 and 100 µg in corresponding wells) respectively in addition to buffer. Phosphate buffer (50 ml, 100 mM, pH 7.0) and fungicide TOPSIN® 4.5 FL (10 µL) both were used as positive and negative controls, respectively. Five technical replicates were done for all concentrations and controls while three measurements were done for each well every time. Fungal growth was observed after 48–72 h of incubation.

The germination rate of the conidia (*F. graminearum*) was checked in 125 µl gene frame chamber (Thermofisher, Catalog, AB0578). Gene frame chamber is perfect for standard microscope slides and they prevent reagent loss during the longer time series. Gene frame chamber contained the conidia in minimal media, *Es*Nap protein (100 µg), BSA (100 µg) and phosphate buffer.

### Cell cytotoxicity assay

The cell survival and proliferation MTT (3-(4, 5-dimethylthiazol-2-yl) -2, 5-diphenyl tetrazolium bromide) assay kit (Millipore, USA) was used for rapid and perceptive quantification of cell proliferation and viability. Briefly, 100 µl (1 × 10^5^) of Huh7 cells were cultured in a 96 wells plate using the Dulbecco’s modified Eagle medium (DMEM) supplemented with 10% fetal bovine serum and 100 IU/ml penicillin and 100 μg/ml streptomycin at 37 °C in a CO_2_ incubator for 24 h. *Es*Nap dilutions 3.12, 6.25, 12.5, 25 and 50 µM were added and the plate was incubated at 37 °C in a CO_2_ incubator for another 24 h and three replications were performed and analyzed for each dilution. After 24 h the medium was removed and 100 µl freshly prepared medium was added along with 10 µl MTT solution (5 mg/ml in PBS) as per manufacturer’s instructions. The plate was again incubated in a CO_2_ incubator at 37 °C for 4 h and after this 0.1 ml DMSO was added to dissolve the formazan crystals in the wells. Mitochondrial succinic dehydrogenase in living cells converts the MTT substance in purple formazan crystals that are insoluble in water. The MTT formazan product was detected by measuring the optical density with a multi-channel plate reading photometer at a test wavelength of 570 nm and a reference wavelength of 620 nm^65^. Cell viability was attained by means of the following formula:

$$\begin{aligned} {\text{Cell}}\,{\text{viability}}\,[\% ] & = ({\text{A}}\_{\text{test}}\,(570\;{\text{nm}}) - {\text{A}}\_{\text{test}}\,(620\,{\text{nm}})/({\text{A}}\_{\text{control}}\,(570\,{\text{nm}}) \\ & \quad - \,{\text{A}}\_{\text{control}}\,(620\,{\text{nm}}))*100. \\ \end{aligned}$$

The IC_50_ (50% inhibitory concentration) value was calculated by non-linear regression analysis with GraphPad Prism software. The assay was conducted in triplicate. One way ANOVA was performed on data with a level of significance *P* < 0.05.

### Entomotoxicity assay

Entomotoxicity assays applying napin were performed in the Eco-toxicology laboratory, Faculty of Agriculture Sciences and Technology, Bahauddin Zakayria University Multan. Napin toxicity was determined for *T. castaneum*. A population of *T. castaneum* was collected from a local flour mill and was cultured on whole wheat flour with 5% brewer yeast^66^. To get an equal age insect population, the culture medium was complete wheat flour incubated at 60–90 °C for 60 min. One glass jar was used and filled with 500 g flour and 50 red flour beetles were added. For the oviposition beetles were left in the culture medium. After three days beetles were removed with the help of sieves and then added to a separate set of sterilized jars filled with 200 g flour for maintenance of the culture. Flour containing eggs was used as culture medium for obtaining adult beetles of a homogenous population^67,68^. The culture was maintained under optimum laboratory conditions at 30 °C with a relative humidity of 70%.

For the bioassays three different serial dilutions of napin were prepared in 100 mM phosphate buffer and 3, 2 and 1 mg/ml protein concentrations were used. A total of 450 g of wheat flour was pre-refrigerated at 4 °C to avoid any infestation. Each protein concentration was prepared in 100 ml buffer solution mixed with 150 g flour to form homogeneous dough. It was dried in the dark to form a hard pan and subsequently grinded with an electric grinder providing powder. Five replications using one fifth of the material each for all three concentrations and a control for comparison, i.e. buffer with no napin, were set up. Each replication was executed in an individual glass jar. Five males and five females of *T. castaneum* were released in each jar. After ten days, released adults were removed and interval data of larvae, male and female pupae including adults were recorded weekly.

### Statistical analysis

The entomotoxin protein bioassay data were analyzed in one way ANOVA through the stat software “Statistix 8.1” and mean values were separated by a Tukey-HSD test with a level of significance of 0.05 (Analytical Software, 2005).

## Conclusions

In conclusion, a napin protein was isolated and purified from *Eruca sativa* and contains disulfide bonds in a monomeric form. Furthermore, the napin inhibits the growth of *F. graminearum* at the stage of conidia and possesses cytotoxicity towards Huh7 cells. Based on its above-mentioned properties, different napins like *Es*Nap may promote the resistance of plants against infections by parasitic fungi and likewise reduce the susceptibility towards other plant pathogens.

## Supplementary Information

Supplementary Information.
