# Supplementary material for: Eruca sativa seed napin structural insights and thorough functional characterization
Source: Sci Rep. 2021 Dec 15;11:24066. doi: 10.1038/s41598-021-02174-6 (PMC8674280; doi:10.1038/s41598-021-02174-6)
Supplement: Supplementary file 1 — Supplementary Information. [file 41598_2021_2174_MOESM1_ESM.docx]

**Supplementary Data**

**
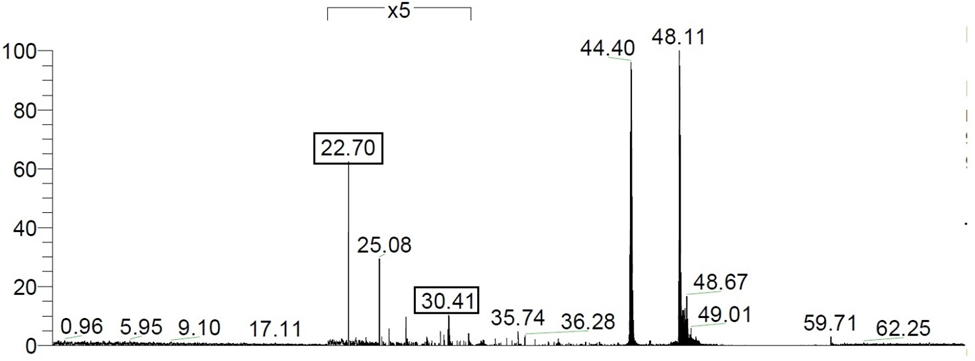
**

**Figure S1:** LC-MS/MS chromatogram of identified peptides from *E. sativa* napin protein tryptic digestion (m/z values are highlighted).


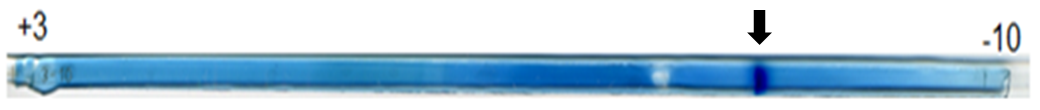


**Figure S2**: Isoelectring focusing of *Es*Nap at pH 7 is indicating a slightly alkaline pI of approximately 8.

**
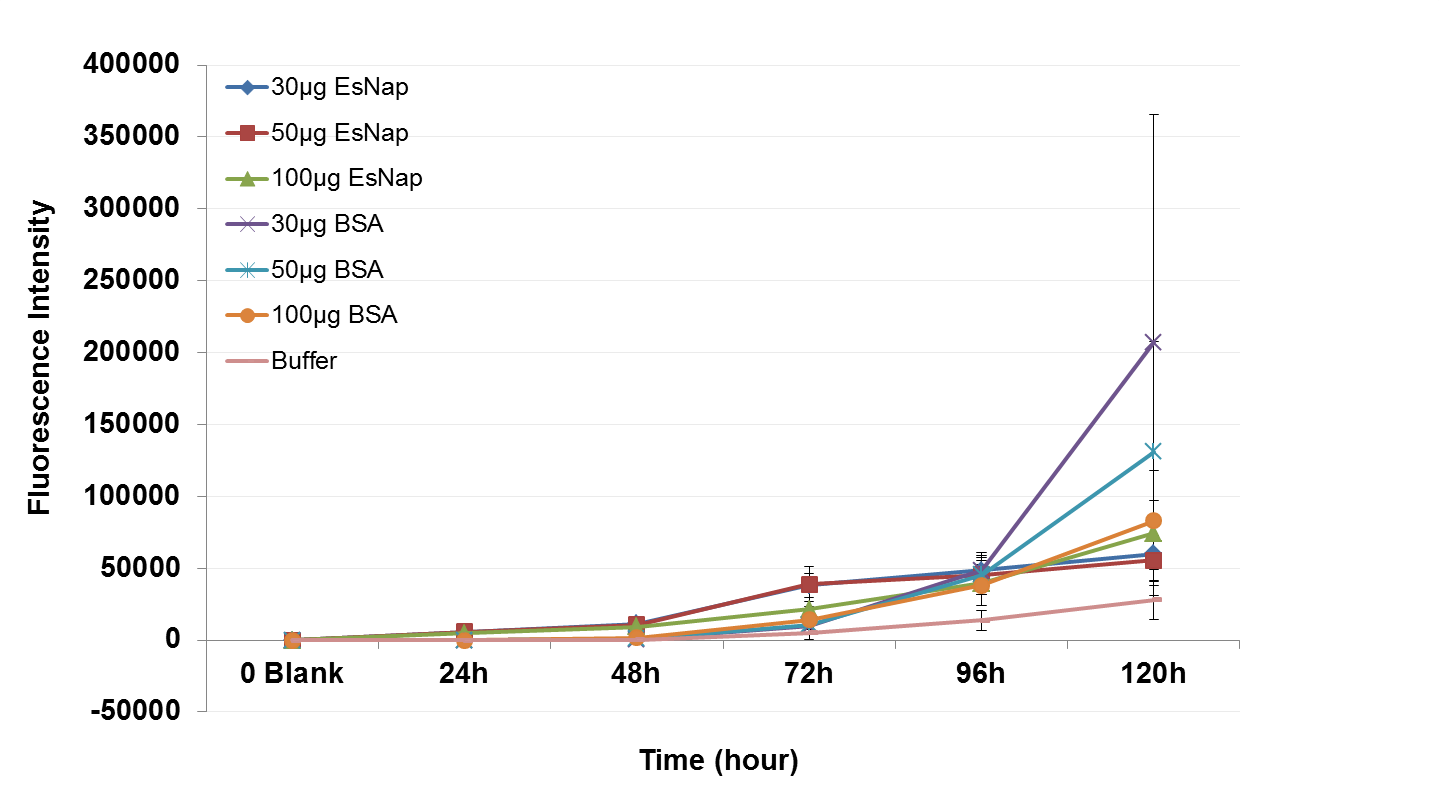
**

**Figure S3:** Antifungal activity of *Es*Nap from 0 h to 120 h growth


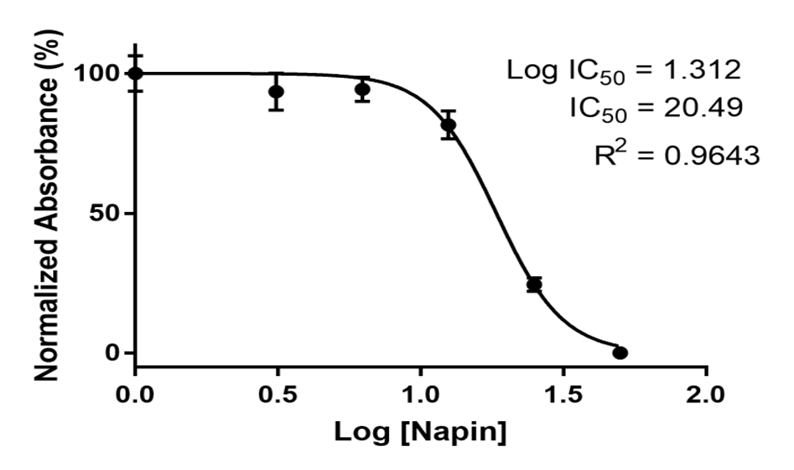


**Figure S4:** Non- linear regression analysis for calculation of *Es*Nap IC_50_. 20.49 µM values indicated the IC_50_.
